# New transperineal ultrasound-guided biopsy for men in whom PSA is increasing after Miles’ operation

**DOI:** 10.1186/s13244-023-01384-y

**Published:** 2023-03-16

**Authors:** Byung Kwan Park, Jae Hoon Chung, Wan Song, Minyong Kang, Hyun Hwan Sung, Hwang Gyun Jeon, Byong Chang Jeong, Seong Il Seo, Seong Soo Jeon, Hyun Moo Lee, Ghee Young Kwon

**Affiliations:** 1 Department of Radiology, Samsung Medical Center, Sungkyunkwan University School of Medicine, 81 Irwon-Dong, Gangnam-Ku, Seoul, 06351 Republic of Korea; 2 Department of Urology, Samsung Medical Center, Sungkyunkwan University School of Medicine, Seoul, Republic of Korea; 3 Department of Pathology, Samsung Medical Center, Sungkyunkwan University School of Medicine, Seoul, Republic of Korea

**Keywords:** Prostate cancer, Miles’ operation, Transperineal US, Biopsy techniques, Biopsy outcomes

## Abstract

**Objectives:**

Currently, a prostate biopsy is guided by transrectal ultrasound (US) alone. However, this biopsy cannot be performed in men without an anus. The aim of this study was to show the outcomes of a new transperineal US (TPUS)-guided biopsy technique in patients who underwent Miles’ operation.

**Methods:**

Between April 2009 and March 2022, TPUS-guided biopsy was consecutively conducted in 9 patients (median, 71 years; range, 61–78 years) with high prostate-specific antigen values (22.60 ng/mL; 6.19–69.7 ng/mL). Their anuses were all removed due to rectal cancer. TPUS-guided biopsy was performed according to information on prostate magnetic resonance imaging. The technical success rate, cancer detection rate, and complication rate were recorded. Tumor sizes were compared between benign and cancer groups using an unpaired t-test with Welch’s correction.

**Results:**

The new TPUS-guided biopsy was successfully performed in all patients. Cancer was detected in 77.8% (7/9) of the patients. These were all categorized as PI-RADS 5. Among them, the detection rate of significant cancer (Gleason score 7 or higher) was 66.7% (6/9). The median tumor size was 2.4 cm (1.7–3.1 cm). However, two patients were diagnosed with benign tissue with PI-RADS 3 or PI-RADS 4. Their median tumor size was 1.0 cm (0.8–1.2 cm). There was significant difference between the cancer and benign groups (*p* = 0.037) in terms of tumor size. Neither post-biopsy bleeding nor infections occurred.

**Conclusions:**

New TPUS-guided biopsy technique may contribute to detecting large PI-RADS 5 prostate cancer in men after Miles’ operation.

## Key points

- The new transperineal ultrasound (TPUS) technique can offer an accessible route in men without an anus.
- A PI-RADS 5 tumor is a good indication for the new TPUS-guided biopsy technique.
- The TPUS-guided biopsy is difficult to target a small prostate tumor (< 1.5 cm).

## Introduction

Ultrasound (US)-guided biopsy is the mainstay for detecting prostate cancer in patients with high prostate-specific antigen (PSA) values [1]. Biopsy cores are sampled via transrectal (TR) or transperineal (TP) approaches. These biopsy techniques cannot be performed without transrectal ultrasound (TRUS) guidance. Because TRUS is not possible in patients without an anus, tissue samples cannot be collected by the current biopsy procedures in such patients,. In addition, a magnetic resonance imaging (MRI)-guided in-bore biopsy is not possible because biopsy cores are sampled via a TR approach, either [1, 2].

A small number of patients who undergo Miles’ operation have a biopsy due to rising PSA values. The new biopsy procedure was performed under TPUS guidance alone. Therefore, we hypothesized that TPUS-guided biopsy could target a prostate tumor. To our knowledge, only a few studies have demonstrated the utility of TPUS-guided biopsy in men without an anus [3, 4]. However, only cases and the accompanying figures were reported. The purpose of this study was to retrospectively assess the technique and outcomes of using the new TPUS-guided biopsy in patients who underwent Miles’ operation.

## Materials and methods

This retrospective study was approved by the Institutional Review Board, and the requirement for informed consent was waived.

### Subjects

Between April 2009 and March 2022, TPUS-guided biopsy was conducted in 9 consecutive patients (median, 71 years; range, 61–78 years) with high PSA values (22.60 ng/mL; 6.19–69.7 ng/mL) (Table 1). Their median prostate volume and PSA density were 26.8 mL (17.4–92.1 mL) and 0.82 ng/mL^2^ (0.07–2.60 ng/mL^2^), respectively (Table 1). Because all patients underwent Miles’ operation due to rectal cancer, a transducer could not be introduced through the anus. The inclusion criteria included a history of Miles’ operation and elevated PSA values (more than 2.5 ng/mL). None of these cases had undergone a prostate biopsy previously. We had only one exclusion criterion: During the same study period, all cases undergoing TP or TR biopsy guided by TRUS were excluded.

**Table 1:** Patient demographics

Patient No	Age	PSA (ng/mL)	Prostate volume (mL)	PSAD (ng/mL^2^)	Biopsy (year)	3 T MRI		TPUS	
						Scanner	Coil	Scanner	Probe
1	71	23.61	21.1	1.12	2009	Achieva (PH)	Surface	IU 22 (PH)	C9 5ec (PH)
2	77	6.19	92.1	0.07	2012	Achieva (PH)	Surface	IU 22 (PH)	C9 5ec (PH)
3	70	11.92	19.4	0.61	2016	Skyra (SH)	Surface	IU 22 (PH)	C9 5ec (PH)
4	72	22.60	19.8	1.14	2017	Achieva (PH)	Surface	IU 22 (PH)	C9 5ec (PH)
5	66	25.29	17.4	1.45	2019	Achieva (PH)	Surface	IU 22 (PH)	C9 5ec (PH)
6	78	22.69	27.8	0.82	2020	Achieva (PH)	Surface	EPIC (PH)	C10 4ec (PH)
7	71	11.70	56.3	0.21	2020	Ingenia CX (PH)	Surface	EPIC (PH)	C10 4ec (PH)
8	64	69.70	26.8	2.60	2021	Ingenia CX (PH)	Surface	EPIC (PH)	C10 4ec (PH)
9	61	10.10	53.0	0.19	2022	Magnetom Vida (SH)	Surface	EPIC (PH)	C10 4ec (PH)

No., number; PSA, prostate-specific antigen; PSAD, prostate-specific antigen density; MRI, magnetic resonance imaging; TPUS, transperineal ultrasound; PH, Philips Healthcare; SH, Siemens Healthcare

### MRI analysis

Prior to the biopsy, prostate MRI using a surface coil was performed in all cases (Table 1). A single radiologist prospectively categorized the index tumor in 7 patients between 2016 and 2022 using prostate imaging reporting and data system (PI-RADS) version 2 or 2.1 (Table 2) [5, 6]. However, MR images, which were acquired in 2 patients from 2009 to 2015, were retrospectively categorized with PI-RADS version 2.1. He was a genitourinary radiologist who had a 7-year experience of MRI interpretation prior to the first TPUS-guided biopsy.

**Table 2:** MRI and biopsy results

Patient No	MRI findings					TPUS-guided biopsy findings			
	Locations		Tumor size (cm)	PI-RADS score	MRI stage	Gleason score	Biopsy cores	Cancer cores	Cancer length (%)
	Transverse	Longitudinal							
1	TZ	Base-Apex	2.4	5	T2c	6 (3 + 3)	4	1	5
2	TZ	Base	0.8	3	T2a	No cancer	4	0	0
3	PZ	Base-Apex	1.7	5	T3a	7 (4 + 3)	4	4	70
4	PZ	Base-Apex	2.1	5	T3a	7 (4 + 3)	1	1	60
5	PZ	Base	2.4	5	T3b	9 (5 + 4)	4	4	30
6	PZ	Base-Apex	3.1	5	T3b	8 (4 + 4)	4	4	20
7	PZ	Base	1.2	4	T2a	No cancer	7	0	0
8	TZ	Base-Apex	2.5	5	T3a	7 (4 + 3)	2	2	100
9	PZ	Base-Middle	2	5	T2c	7 (3 + 4)	1	1	80

No., number; TZ, transition zone; PZ, peripheral zone; PI-RADS, Prostate Imaging Reporting and Data System; MRI, magnetic resonance imaging; TPUS, transperineal ultrasound. Cancer length indicates what percentage the tumor cells occupied in the biopsy core with the highest Gleason score

**Fig. 1:**
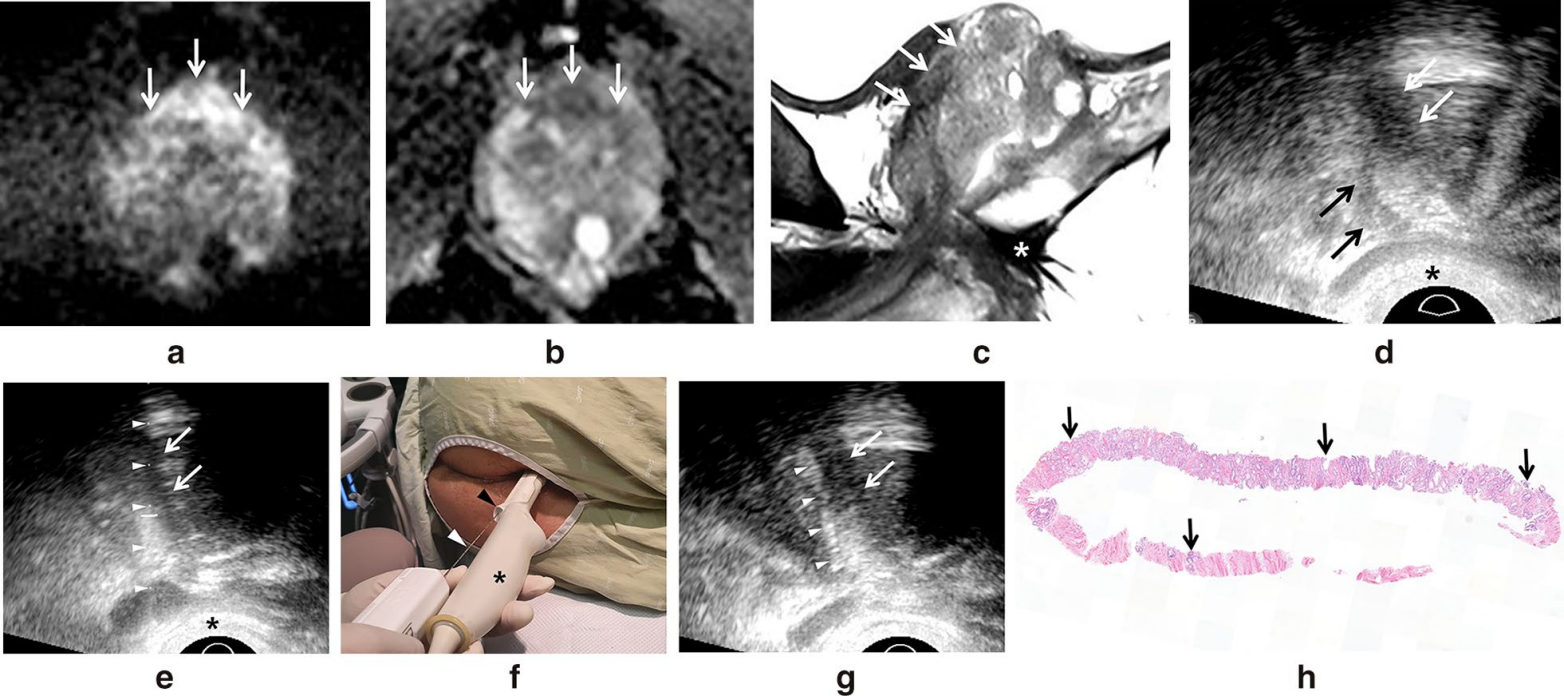
A 61-year-old man (case 9) with a high PSA value. **a** Diffusion-weighted axial image (b value = 1400 s/mm) shows a 2.0 cm hyperintense mass (white arrows) at the anterior midline base. The patient underwent Miles’ operation due to rectal cancer in 2011. Recently, his PSA was as high as 10.10 ng/mL. **b** Apparent diffusion coefficient (ADC) map axial image shows the tumor (white arrows) has low ADC values. The tumor was consistent with PI-RADS 5. **c** T2-weighted sagittal image shows that the tumor (white arrows) is located from base to midgland. The white asterisk indicates the absent rectum and anus. **d** Longitudinal TPUS image shows a hypoechoic tumor (white arrows). The black arrows indicate the prostate urethra in the path of the biopsy needle. The black asterisk indicates subcutaneous fat compressed by the transducer. **e** Longitudinal TPUS image shows a guideline (white arrowheads) for creating a needle path. It is placed in the center of the tumor (white arrows). However, it does not cross the prostate urethra to avoid injury. The black asterisk indicates subcutaneous fat compressed by the transducer. **f** The photograph shows a biopsy needle (black arrowhead) being introduced into the guider (white arrowhead) on the transducer (black asterisk). **g** Longitudinal TPUS image shows the hyperechoic path (white arrowheads) of the biopsy needle targeting the posterior aspect of the tumor (white arrows). However, it did not cross the prostate urethra. Only one core was obtained to avoid urethra injury. **h** Hematoxylin and eosin-stained slide shows the length (black arrows) of the cancer tissue occupying 80% of the entire core. GS 7 (3 + 4) was confirmed by a genitourinary experienced pathologist

The PI-RADS scores ranged from 3–5 (median, 5). Tumor sizes were measured on T2-weighted or diffusion-weighted MR images. The median size of the index tumors was 2.1 cm (range, 0.8–3.1 cm). The transverse locations of the index tumors were the peripheral zone in 6 and the transition zone in 3 patients (Fig. 1a, b, and c). The longitudinal locations were base-to-apex in 5, the base in 3, and base-to-mid-gland in one patient (Fig. 1a, b, and c). The tumor stages on MRI were T2a in 2, T2c in 2, T3a in 3, and T3b in 2 patients.

### Biopsy procedures

All patients fasted for 6 h. They took oral antibiotics from one day before the biopsy to 6 days post-biopsy. All patients were laid in a left decubitus and knee-chest position. An end-fire endo-cavity transducer (5–9 MHz or 4–10 MHz) was placed in contact with the perineum by the same radiologist who had interpreted the pre-biopsy MR images (Table 1) and had 11 years of experience with prostate biopsies prior to the first case 2009. First, the prostate urethra was searched as a landmark in the sagittal planes by pushing a transducer to the perineum (Fig. 1D). Then, the radiologist scanned the entire prostate to localize the index tumor detected on MRI (Fig. 1E). After tumor localization, the perineum was sterilized with alcohol and anesthetized with a total of 5–10 mL of 2% lidocaine, which was repeatedly injected along the biopsy path until the patient no longer complained of pain. An 18-gage automated co-axial needle (ACECUT; TSK Laboratory, Tochigi-shi, Japan) was introduced into a guider placed on the transducer (Fig. 1F). The needle tip was placed as close to the index tumor as possible. All biopsy cores were multi-focally sampled within the tumor except for two cases in which one core was obtained. The biopsy tracts were easily detected because they were mostly hyperechoic in the TPUS images (Fig. 1G).

### Pathologic examination

All biopsy cores were examined by an experienced pathologist who had been dedicated to genitourinary pathology for 13 years prior to the first case. He determined whether each core was positive or negative for cancer and recorded the Gleason scores or the length of the cancer tissue within the entire positive core as a percentage. His assessment was based on the 2005 or 2014 International Society of Urological Pathology Consensus Conference guidelines [7, 8].

### Statistical analysis

An unpaired t-test with Welch correction was performed to compare tumor sizes between the cancer and benign groups. Commercially available SPSS 24.0 software for Windows (SPSS Inc., Chicago, IL, USA) was used for the statistical analyses. A *p*-value of < 0.05 was considered statistically significant.

## Results

Cancer was detected in 77.8% (7/9) of the patients. These were all categorized as PI-RADS 5 (Table 2). Among them, a GS of 6 (3 + 3) was confirmed in one patient. Of the remaining 6 patients, one had GS 7 (3 + 4), 3 had GS 7 (4 + 3), one had GS 8 (4 + 4), and one had GS 9 (5 + 4). The median tumor size was 2.4 cm (1.7–3.1 cm), and the tumor locations were the base in one patient, base-to-middle in one, and base-to-apex in 5 patients. The positive core rate was 54.8% (17/31) in all patients and 85.0% (17/20) in the cancer patients. The median cancer length was 60% (5–100%) (Fig. 1H). In contrast, no cancer cells were detected in 2 patients who had PI-RADS 3 or PI-RADS 4. The median tumor size was 1.0 cm (0.8–1.2 cm), and the tumor locations were all at the base. The tumor sizes of these patients were significantly smaller than those of the cancer-proven patients (*p* = 0.037). None of the patients experienced post-biopsy bleeding or infections.

## Discussion

Our results showed that the new TPUS procedure helped radiologists to target prostate tumors in men without an anus due to prior Miles’ operation. All PI-RADS 5 lesions were technically successful in detecting cancer cells. However, this new biopsy technique did not sample cancer cores from small PI-RADS 4 or lower lesions.

Miles’ operation is necessary for patients with rectal cancer that is too close to secure a sufficient resection margin [9, 10]. Accordingly, their anuses should be removed to avoid marginal recurrence. However, an absent anus poses challenges to radiologists or urologists in performing prostate biopsies in patients with increasing PSA values. A TRUS-guided biopsy cannot be performed because a transducer cannot be introduced into the patient’s absent anus. TP biopsy is also impossible because TRUS is essential for guiding it. Thus, the current prostate biopsy procedures are technically not possible in patients with prior Miles’ operation.

TPUS is a useful examination for assessing various diseases in the perineum, which is superficially located from the skin. This examination is frequently used in women who have inflammation, infections, or tumors arising from the vagina, urethra, or anus [11–13]. Generally, a linear array transducer is applied for lesion detection in these situations, but it has a limitation that its US does not reach deep-sited organs. Originally, a TR transducer was designed for introduction into the rectum. It produces 4–10 MHz US waves, but the mean frequency is slightly lower than that of a linear-array transducer. Therefore, a TR transducer can assess organs deeper-sited from the skin. In addition, Miles’ operation moves the prostate closer to the perineum through the removal of the rectum and peri-rectal fat. Accordingly, the shorter distance between the skin and the prostate helps in detecting prostate cancer through TPUS.

MR images should be assessed thoroughly by the radiologist who will perform the biopsy. They need to identify the size, location, and shape of an index tumor and precisely categorize it based on PI-RADS version 2.1. They should also be familiar with the different scan axes between MRI and TPUS. MRI is scanned perpendicularly to the urethra, but TPUS is performed obliquely to it. Like TRUS [3, 14, 15], as a tumor approaches the anterior capsule on MRI, it is located more inferiorly on TPUS. In contrast, when a tumor is closer to the posterior capsule on MRI, it is located more superiorly on TPUS [3, 14, 15]. Moreover, as a tumor becomes significant, peripheral cancer becomes hypoechoic compared to normal peripheral tissue, whereas transition cancer becomes hyperechoic compared to hyperplastic tissue [14–16]. In addition, adopting fundamental US and a low dynamic range will help to improve the contrast between significant cancer and normal tissue [14]. This information on imaging protocols and findings is of great importance for the precise targeting of TPUS, as well as TRUS [17].

Radiologists or urologists should be familiar with the technical tips of TPUS-guided biopsies. A transducer should be pushed to the perineum to be closer to the prostate and can easily detect and target an index tumor because of its better image quality [3, 4]. The prostate urethra should be identified because it is a good landmark to guide a scan and biopsy. Lidocaine injections should be repeated to control pain as much as possible. Sufficient local anesthesia is a key procedure for patient cooperation. Monitored anesthesia care, spinal anesthesia, or general anesthesia is frequently performed to obtain many cores in the current TP biopsy procedure guided by TRUS [18, 19]. However, local anesthesia is enough for pain control in our TPUS-guided biopsy because it worked well in all cases. The entire pathway of the biopsy needle approaching the index tumor must be attended to and carefully visualized to avoid injury to the prostate urethra or peri-prostatic vessels. Frequently, hyperechoic lines seen within the tumor are good imaging features for precise targeting [14]. As soon as TPUS-guided biopsy is completed, color Doppler US is performed to determine if there is active bleeding [20]. TPUS-guided compression is a useful way to reduce the amount of acute hematoma. It should be applied to the perineum until the bleeding resolves.

This study had several limitations. First, the number of cases was small because the incidence is not high. This weakness is the small sample size which is expected because of the limited patient population that the inclusion criteria apply to. Second, radiologists or urologists may need a long time to become familiar with TPUS features and biopsy skills for precise targeting. Third, TPUS provides relatively poor image quality compared to TRUS. The distance between the transducer and the prostate is longer in TPUS than in TRUS. The TPUS transducer gives off US waves from the perineum to depict the prostate. In contrast, the TRUS transducer is introduced into the endo-rectal cavity, and operators push it to the prostate. As a result, the transducer-to-prostate distance is much shorter on TRUS, resulting in better image quality. A limitation of TPUS-guided biopsy is poor depiction of a prostate lesion. Therefore it is not surprising that no cancer was detected for the smaller tumors. In terms of applicability to clinical practice, TPUS-guided biopsy may be a good option for larger tumors and high clinical suspicion of prostate Ca, but these are typically higher stage and/or may be locally advanced.

Third, poor US visibility is also a risk factor for urethral injury although none was encountered in this study. Our study is a good proof of concept but larger scale studies or further validation is needed.

## Conclusion

New TPUS techniques can help radiologists or urologists produce an accessible biopsy route to the prostate, even when the patient’s anus is resected by Miles’ operation. A large tumor categorized as PI-RADS 5 is a good indication for the new biopsy technique. However, this TPUS-guided biopsy may not target a small tumor of less than 1.5 cm. Therefore, operators need to be familiar with the new TPUS-guided biopsy procedure.

## Data Availability

The data presented in this study are available upon request from the corresponding author.

## Abbreviations

GS: Gleason score
PI-RADS: Prostate Imaging Reporting and Data System
PSA: Prostate-specific antigen
TPUS: Transperineal ultrasound
TRUS: Transrectal ultrasound
